# Genome-Wide Identification and Functional Analysis of the *PEBP* Gene Family in *Begonia semperflorens* ‘Super Olympia’ Reveal Its Potential Role in Regulating Flowering

**DOI:** 10.3390/ijms26136291

**Published:** 2025-06-29

**Authors:** Congcong Fu, Mengru Zhao, Huiting Xia, Puyu Ren, Weichao Liu, Qirui Wang, Kaiming Zhang

**Affiliations:** College of Landscape Architecture and Art, Henan Agricultural University, Zhengzhou 450002, China; 15314532106@163.com (C.F.); 13213180216@163.com (M.Z.); xhuiting223@163.com (H.X.); renpuyu736@163.com (P.R.); liuweichao@henau.edu.cn (W.L.); wangqr@henau.edu.cn (Q.W.)

**Keywords:** *PEBP* gene, *B. semperflorens* ‘Super Olympia’, flowering

## Abstract

The phosphatidylethanolamine-binding protein (PEBP) gene family, known for its pivotal role in controlling floral transition, regulates flowering time, and, thus, shapes the continuous-flowering trait in ornamental plants. In this study, we conducted the first genome-wide identification and bioinformatics analysis of the *PEBP* gene family in *Begonia semperflorens* ‘Super Olympia’, a variety that exhibits year-round flowering. Via phylogenetic analysis, a total of 10 *BsPEBP* genes were identified and categorized into four subfamilies: the FT-like (two members), TFL1-like (three members), PEBP-like (three members), and MFT-like (two members) subfamilies. Gene structure analysis revealed highly conserved motif compositions among family members, and protein tertiary structure prediction indicated the dominance of random coils in their structures. Promoter cis-acting element analysis revealed light-responsive, hormone-responsive (ABA, GA, and JA), and abiotic stress-responsive elements in the *BsPEBP* genes, suggesting their potential integration into multiple regulatory pathways. The tissue-specific expression profiles revealed that *BsPEBP6* was significantly upregulated in floral organs, whereas TFL1-like subfamily members were predominantly expressed in vegetative tissues. These findings imply that the *FT-like* and *TFL1-like* genes antagonistically regulate the continuous-flowering trait of *B. semperflorens* ‘Super Olympia’ through their respective roles in promoting and repressing flowering. Our findings provide a preliminary theoretical foundation for elucidating the molecular mechanisms by which the *PEBP* gene family regulates flowering time in ornamental plants and offer valuable insights for developing breeding strategies aimed at flowering time modulation.

## 1. Introduction

Genes of the phosphatidylethanolamine-binding protein (PEBP) family are found in all three major phylogenetic divisions, that is, bacteria, archaea, and eukaryotes [1,2,3] and in plants, they play important roles in regulating floral transition and seed germination [4,5,6]. The *PEBP* gene family in plants, members of which serve as critical regulators of floral transition and plant architecture establishment, has become a research hotspot in plant developmental biology because of its functional diversification and molecular evolutionary mechanisms [7]. Phylogenetic analyses have revealed that this family can be divided into three functional subgroups: FT, TFL1, and MFT. Among these genes, the *FT-like* genes *AtFT* and *SFT* act as florigen-encoding genes that promote flowering. Under conditions of extended daylight, the expression of FT is induced, resulting in the formation of the FT-FD complex with FD. This complex then activates the expression of the *APETALA1* (*AP1*) and *LEAFY* (*LFY*) genes, thereby facilitating the transition to the flowering stage [8,9]. Recent studies have identified several regulatory pathways associated with flowering: the photoperiod, temperature-sensitive, vernalization, autonomous, hormone, and age-related pathways [10,11]. Unlike FT, TFL1 inhibits the plant transition from the inflorescence meristem to the floral meristem, thus delaying flowering [12,13]. In Arabidopsis sp., *TFL1* regulates the meristem genes *LEAFY* (*LFY*) and *AP1* to control the plant’s morphological structure [14,15]. In contrast, the functional characterization of the MFT-like subgroup remains incomplete, with recent studies suggesting potential roles of these genes in the regulation of seed germination [4] and redundant floral induction and governing the process of seed germination via the abscisic acid (ABA) and gibberellin (GA) signaling pathways in *Arabidopsis*. Recent studies have highlighted the central role of the PEBP family in continuous-flowering-related traits [16]. The overexpression of *PgTFL1* and *PgCENa* in pomegranate (*Punica granatum*) was able to suppress the flowering defect of the *Arabidopsis* tfl1-14 mutant [17]. In rice (*Oryza sativa*), *OsCEN* and *Hd3a* [18] (*heading date 3a*, an ortholog of the FT protein) competitively bind to *OsFD* and regulate flowering [13]. The *TFL1-like* gene is downregulated in adult *D. catenatum*, suggesting that a mechanism inhibits flowering during the vegetative growth phase in rattan plants [19]. The PEBP family has been identified in various plants, such as bamboo (gene number [n] = 6) [20], *Oryza sativa* (n = 19) [10], *Gossypium hirsutum* (n = 8) [21], common wheat (n = 76, 38, 16, and 22) [22], *Glycine max* (n = 23) [23], *Vitis cinerea* (n = 5) [24], Rosaceae tree species (n = 56) [25], and maize (n = 25) [26]. The PEBP family in the important ornamental variety *B. semperflorens* ‘Super Olympia’, known for its continuous flowering trait, has yet to be systematically characterized. This study examines the unique continuous-flowering trait of *B. semperflorens* ‘Super Olympia’ by conducting the first genome-wide identification and bioinformatics analysis of the *BsPEBP* gene family. Through systematic exploration of evolutionary relationships, gene structures, and expression regulatory features of PEBP family members, we aimed to uncover their potential associations with year-round flowering. The results provide a theoretical foundation for elucidating the BsPEBP regulatory network, elucidating the molecular mechanisms of floral transition, and laying the groundwork for flowering time regulation in ornamental plant breeding.

## 2. Results

### 2.1. Identification of PEBP-like Genes in B. semperflorens ‘Super Olympia’

Through homologous sequence alignment, this study identified a total of 10 *B. semperflorens* ‘Super Olympia’ *PEBP* genes (Table S1), designated *BsPEBP1-BsPEBP10*. Physicochemical analysis revealed that BsPEBPs contain 112–182 amino acids (aa), with theoretical isoelectric points (pIs) ranging from 4.98 to 9.33. Most BsPEBPs are classified as basic proteins. Among them, *BsPEBP2*, *BsPEBP3*, *BsPEBP5*, and *BsPEBP8* presented instability indices below 40, whereas the remaining proteins presented instability indices exceeding 40, suggesting that most BsPEBPs are unstable. All of the BsPEBPs had a grand average of hydropathicity (GRAVY) values below 0, indicating that they are hydrophilic proteins. Subcellular localization predictions revealed that three BsPEBPs localize to the cytoplasm and five to the nucleus, and *BsPEBP8* and *BsPEBP10* were predicted to localize to both the nucleus and the cytoplasm.

### 2.2. Sequence Alignment and Phylogenetic Relationship Analysis

**Figure 1:**
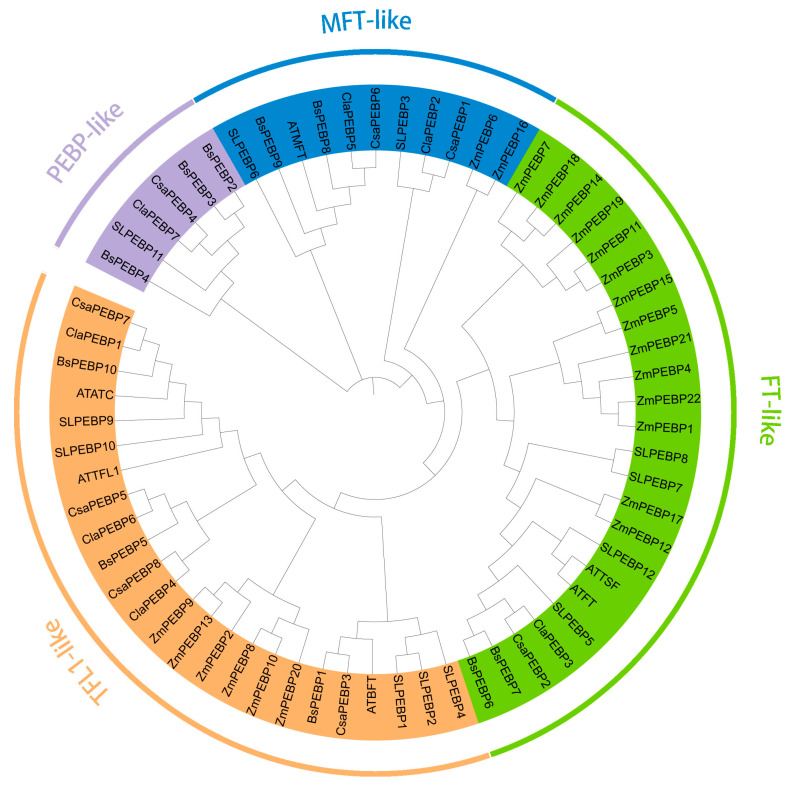
Phylogenetic tree of PEBPs in *Arabidopsis thaliana*, *Solanum lycopersicum*, *Zea mays*, *B. semperflorens* ‘Super Olympia’, *Citrullus lanatus*, and *Cucumis sativus*. Note: at: *Arabidopsis thaliana*; SL: *Solanum lycopersicum*; Zm: *Zea mays*; Bs: *B. semperflorens* ‘Super Olympia’; Csa: *Cucumis sativus;* Cla: *Citrullus lanatus*.

To clarify the phylogenetic relationships of the *B. semperflorens* ‘Super Olympia’ PEBPs, sequence alignment was conducted (Figure S1), and a phylogenetic tree was constructed using PEBPs from the model organisms *Arabidopsis thaliana*, *Solanum lycopersicum*, *Zea mays*, and *Cucurbitaceae* family (watermelon, cucumber) that are closely related to the *Begoniaceae* family (Figure 1). The results revealed that 65 PEBPs from these species clustered into four subfamilies, FT-like, MFT-like, PEBP-like, and TFL1-like, and sequence alignment revealed that proteins from different species within the same subfamily presented sequence similarity (Figure S2). The FT-like subfamily, comprising 24 members, included 2 *Arabidopsis FT* homologs (*ATTF* and *ATTSF*), 4 tomato FT-like proteins, 14 maize PEBPs, 1 watermelon *PEBP*, 1 cucumber *PEBP*, and 2 *B. semperflorens* ‘Super Olympia’ *PEBP* homologs. The MFT-like subfamily contained 11 members: *Arabidopsis MFT*, 2 tomato *MFT* homologs, 2 maize *PEBPs*, 2 watermelon *PEBPs*, 2 cucumber *PEBPs*, and 2 *B. semperflorens* ‘Super Olympia’ *PEBP* homologs. The TFL1 subfamily, which included 24 members, such as *Arabidopsis BFT*, *TFL1*, and *ATC*; 7 tomato homologs; 6 maize *PEBPs*; 3 watermelon *PEBPs*, 3 cucumber *PEBPs*, and 5 *B. semperflorens* ‘Super Olympia’ *PEBP* homologs. The PEBP-like subfamily contained six members: one watermelon *PEBP*, one cucumber *PEBP*, one tomato *PEBP*, and three *B. semperflorens* ‘Super Olympia’ *PEBP* homologs. This phylogenetic analysis highlights the evolutionary conservation and divergence of *PEBP* genes across species, with *B. semperflorens* ‘Super Olympia’ retaining functional homologs in all four subfamilies.

### 2.3. Gene Structure, Conserved Domains, and Motif Analysis

**Figure 2:**
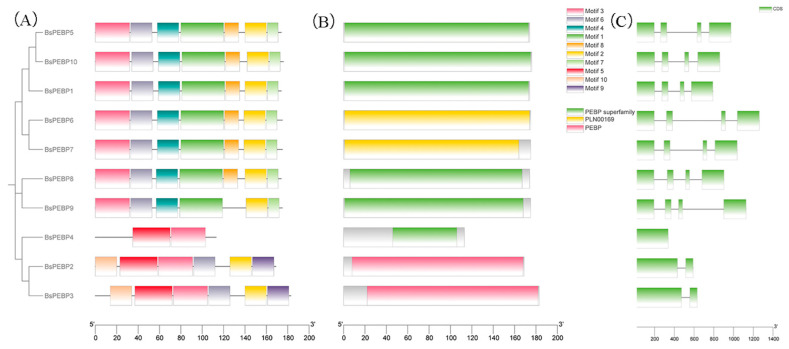
Phylogenetic connections and structural characteristics of the 10 *BsPEBP* genes. Motif composition of *BsPEBP*. The conserved motif number in the MEME prediction was set to nine. Note: (**A**) Phylogenetic tree; (**B**) distribution of conserved motifs; (**C**) structures of PEBP family genes in *B. semperflorens* ‘Super Olympia’. The green regions represent coding sequences (CDSs).

Phylogenetic analysis revealed that the 10 *PEBP* genes in *B. semperflorens* ‘Super Olympia’ can be classified into four subfamilies: FT (two members), TFL1 (three members), PEBP (three members), and MFT (two members) (Figure 2A). This subfamily distribution is consistent with known angiosperm PEBP family patterns, suggesting strong evolutionary conservation. To further investigate functional conservation, we analyzed conserved motifs via MEME. All 10 *BsPEBPs* presented highly similar motif compositions, with most members exhibiting identical motif arrangements (Figure 2B). This minimal divergence underscores the strong purifying selection experienced by these genes. Structural comparisons further validated this conservation. Like Arabidopsis *FT* and *TFL1* homologs, *BsPEBP* genes uniformly possess a four-exon architecture (Figure 2C), indicating evolutionary constraints on splicing patterns. However, an exception emerged in the TFL1-like subfamily: *BsPEBP7* displayed unique intron retention events that were absent in orthologs such as rice *OsCEN* and maize *ZmTFL1*. This structural novelty suggests potential regulatory diversification following whole-genome duplication (WGD) events. Phylogenetic reconstruction provided an evolutionary context. The *BsTFL1-like* genes clustered within a monocot–dicot divergent clade (Figure 1), which was distinct from the core *TFL1* lineages. This lineage-specific clustering, coupled with the unique intron retention of *BsPEBP7*, suggests the occurrence of neofunctionalization events driven by ecological adaptation, possibly to shaded habitats where *B. semperflorens* ‘Super Olympia’ thrives.

### 2.4. Prediction of the Protein-Protein Interaction (PPI) Network

**Figure 3:**
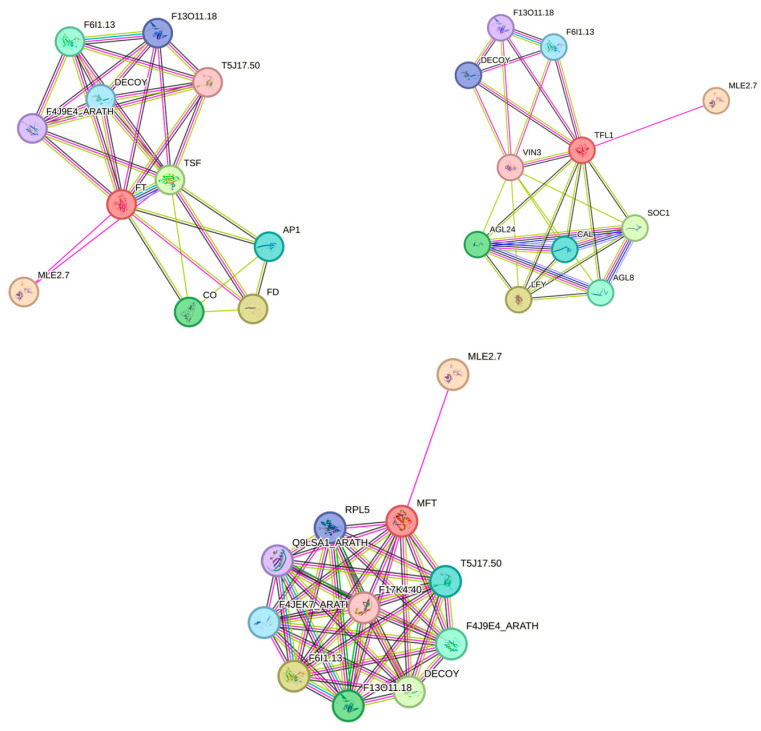
Protein-protein interaction network. The PPI network was predicted on the basis of homologous proteins in *Arabidopsis*. Protein interaction networks of candidate differentially expressed PEBP genes and flowering-regulatory genes.

Phylogenetic comparison of amino acid sequences aids in understanding functional similarity. The amino acid sequence identity between *BsFTs* and *AtFTs* is 86.57%, between *BsMFTs* and *AtMFT* is 81.61%, whereas that between *BsTFL1s* and *AtTFL1* is 74.74% (Figure S3). The sequence identity and conservation of key motifs provide an important foundation for protein-protein interaction (PPI) network prediction, enabling a more intuitive understanding of their functions. STRING analysis revealed that *BsFT* interacts with *TSF*, *CO*, *AP1*, and *FD*, whereas *BsTFL1* interacts with *GAL*, *SOC1*, and *LFY* (Figure 3). The protein interaction prediction results indicate that FT-like, TFL1-like, and MFT-like all interact with *DECOY* and *MLE2.7*, thus suggesting that FT-like, TFL1-like, and MFT-like exhibit antagonistic effects and collectively regulate flowering in *B*. *semperflorens* ‘Super Olympia’. These findings suggest that *BsFTs*, *BsTFL1s,* and *BsMFTs* may perform distinct functions in regulating flowering through different pathways.

### 2.5. Analysis of Promoter Cis-Acting Elements

**Figure 4:**
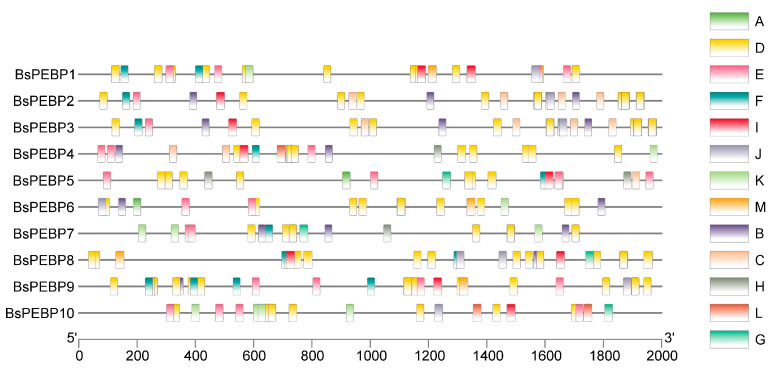
Cis-elements in the *BsPEBP* gene promoter regions. (A) Cis-acting element involved in abscisic acid responsiveness; (B) cis-acting regulatory element involved in MeJA responsiveness; (C) MYB binding site involved in drought inducibility; (D) cis-acting regulatory element involved in light responsiveness; (E) cis-acting regulatory element essential for anaerobic induction; (F) cis-acting regulatory element involved in low-temperature-responsive genes; (G) cis-acting regulatory element related to meristem expression; (H) cis-acting regulatory element related to meristem expression; (I) cis-acting element involved in defense and stress responsiveness; (J) gibberellin-responsive element; (K) auxin-responsive element; (L) cis-acting regulatory element involved in zein metabolism regulation; (M) cis-acting regulatory element involved in circadian control.

For understanding the potential roles and expression regulatory mechanisms of *BsPEBP* genes, cis-acting elements located within the gene promoter regions (upstream 2 kb regions) were predicted using the PlantCARE website (Figure 4). In the promoter region of the *BsPEBP* genes, multiple cis-acting elements were identified and classified into five functional categories: hormone-related elements, including abscisic acid-responsive elements (ABREs) and methyl jasmonate-responsive elements; stress-responsive elements, such as anaerobic-inducible elements and drought-inducible elements; growth- and development-related elements, encompassing meristem-specific elements; light-responsive elements; and transcriptional regulatory elements. ABREs, MeJA response elements, and growth hormone response elements were present mostly in the members of the MFT-like and TFL1-like subfamilies. Light-responsive cis-acting elements were also identified. Additionally, auxin-responsive elements, gibberellin-responsive elements, and salicylic acid-responsive elements were detected (Table S2). These findings suggest that *BsPEBP* genes may integrate regulatory networks linked to growth, developmental processes, and stress responses to mediate their biological functions.

### 2.6. Prediction of Protein Secondary and Tertiary Structure

**Figure 5:**
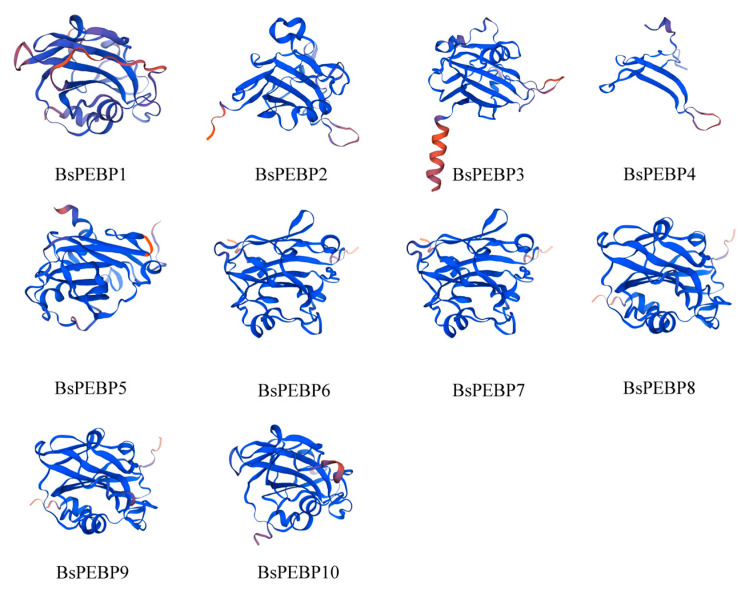
Spatial conformation of the tertiary structures of the BsPEBPs. The tertiary structures of the proteins are composed predominantly of random coils.Dark blue represents a higher level of confidence. Orange represents a lower level of confidence.

The secondary structures of 10 *BsPEBPs* were predicted via the SOPMA online tool. These proteins contain a high proportion of random coils (79.46%) and lack beta turns (Table S3), indicating that their secondary structures are composed predominantly of random coils, with alpha helices and extended strands distributed throughout the proteins. The three-dimensional structures of the proteins were predicted via homology modeling with the SWISS-MODEL online software, (accessed on 7 March 2025). This finding demonstrates that the tertiary structures are also primarily composed of random coils, which is consistent with the secondary structure predictions (Figure 5). The distinct secondary or tertiary architectures of BsPEBPs directly determine their functional divergence. FT-like proteins (BsPEBP6 and BsPEBP7) exhibit dominant random coils conferring conformational plasticity that facilitates allosteric activation: under inductive photoperiods, reversible coil-to-helix transitions expose conserved binding motifs, enabling florigen complex nuclear import. Conversely, TFL1-like member (BsPEBP1) may adopt rigid alpha helices, stabilizing repression complexes through steric occlusion of flowering promoters. Critically, this structural dichotomy enables environmental signal decoding: FT-like intrinsic disorder permits thermosensitive folding for temperature-responsive flowering, while TFL1-like rigidity sustains JA-mediated floral suppression under resource stress. Such structure-function codetermination illustrates how conserved PEBP folds evolved context-dependent regulatory mechanisms in *B. semperflorens’* ‘Super Olympia’ adaptation to shaded niches.

### 2.7. Tissue Expression Patterns of BsPEBP Genes

**Figure 6:**
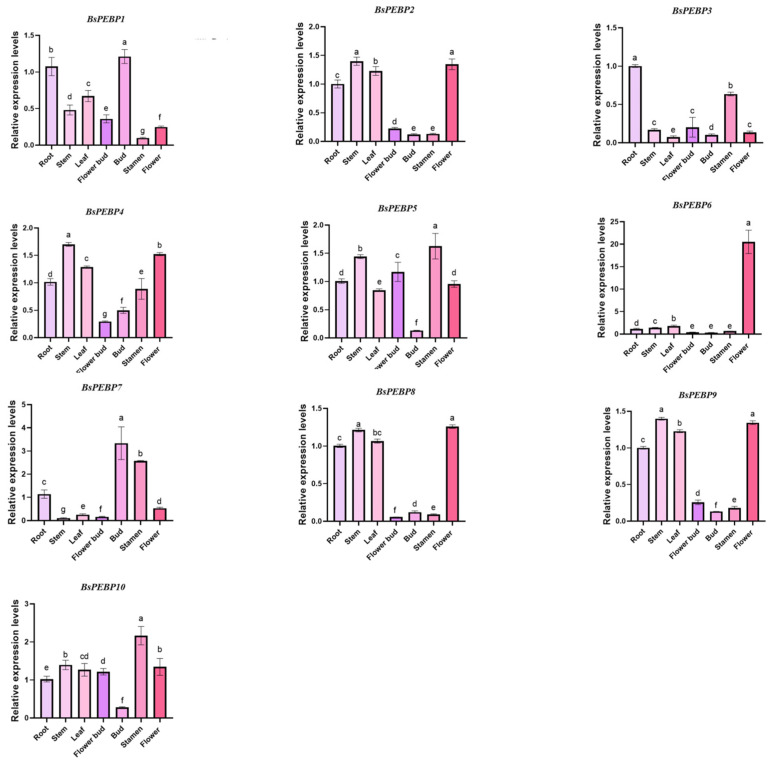
Tissue-specific expression patterns of *BsPEBP* genes. Each test was repeated three times. The error bars in the figure represent the standard deviation. Different letters (a, b, c, d, e, f, and g) in the same column represent statistically significant differences at *p* < 0.05.

Gene functions are intricately tied to spatiotemporal expression dynamics. To elucidate the functional diversification of *BsPEBP* genes, we quantified their spatial expression profiles across seven tissues: roots; stems; leaves; flower buds (not fully developed and elongated); buds (the part of a plant’s flower that has not yet fully opened); stamens and flowers (those in full bloom or fully bloomed) via qRT-PCR (Figure 6). The analysis revealed pronounced tissue-specific divergence, with 40% of the detected members (4 genes) preferentially expressed in stems or leaves, which is consistent with the established role of *PEBP* genes in photoperiodic flowering regulation through transcriptional networks localized to these organs. Subfamily-specific specialization was evident: the *FT-like* gene *BsPEBP6* exhibited floral organ-specific overexpression, whereas its paralog *BsPEBP7* showed bud-specific enrichment. This spatial dichotomy—traditionally linked to reproductive development versus a novel vegetative niche—suggests subfunctionalization through regulatory decoupling, potentially enabling *BsPEBP7* to repurpose ancestral flowering signals for bud-mediated environmental adaptation. In contrast, the *TFL1-like* gene *BsPEBP5* displayed stamen-specific expression, deviating from the canonical shoot apical meristem localization of *AtTFL1* in *Arabidopsis*. This divergence implies lineage-specific evolution of antagonistic FT-TFL1 interactions, where expression domain segregation (stem vs. floral tissues) may establish counterbalancing regulatory modules to fine-tune developmental transitions. Notably, *BsPEBP3* is specifically expressed in root tissues. Analysis of the *BsPEBP3* promoter elements revealed that it contains MYB-binding sites involved in drought-inducible elements. It is speculated that MYB transcription factors can bind to their promoter, activating *BsPEBP3* expression. This activation enhances drought tolerance through the ABA pathway, suggesting its potential role in stress responses. Therefore, the root-specificity appears to be an adaptation that localizes this key stress-responsive function to the primary site of water and nutrient uptake and a critical frontline for sensing soil-borne stresses like drought, a functional innovation distinct from the ancestral roles of *PEBPs* in flowering regulation, analogous to the *OsMFT1*-mediated drought response via ABA signaling in *O. sativa*. Comparative evolutionary analysis highlighted both conservation and divergence. Like *Arabidopsis*, where *FT*-*TFL1* antagonism regulates floral transition, *BsPEBP6* (FT-like) and *BsPEBP5* (TFL1-like) exhibit mutually exclusive expression domains, preserving the core logic of angiosperm flowering regulation. However, *B. semperflorens* ‘Super Olympia’ deviates from seasonal-flowering models through its perennial flowering habit, likely stemming from attenuated TFL1-mediated repression or enhanced FT-TFL1 interaction plasticity—a mechanism mirroring the continuous flowering observed in roses. These adaptations may reflect selective pressures for prolonged reproductive cycles in shaded understory ecologies, where *BsPEBP1* (specifically expressed in flower buds) optimizes reproductive efficiency through the tripartite regulation of organ differentiation, resource allocation, and flowering signal integration.

## 3. Discussion

**Figure 7:**
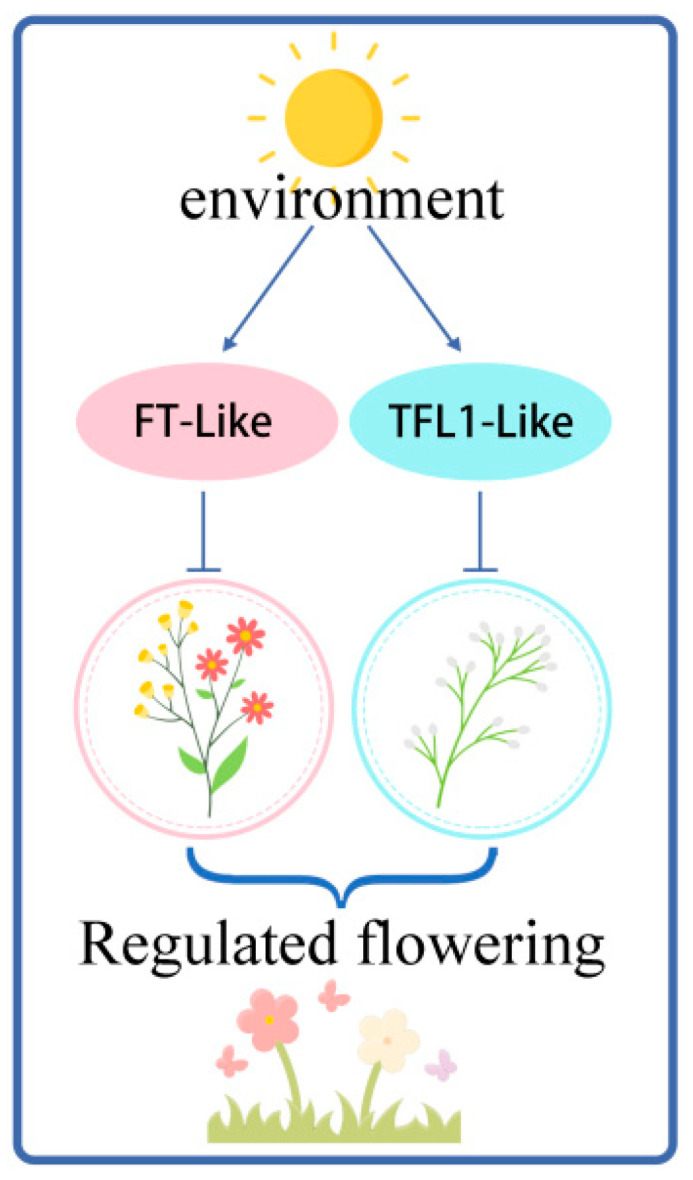
Proposed model for flowering regulation in *B. semperflorens* ‘Super Olympia’.

The PEBP gene family, which is a central regulator of flowering transitions in plants, governs the trade-off between vegetative and reproductive growth, with profound implications for crop yield and ornamental traits [7]. In this study, we performed the first genome-wide identification of *PEBP* genes in *B. semperflorens* ‘Super Olympia’—a horticulturally significant variety exhibiting continuous flowering, identifying 10 *BsPEBP* genes. Phylogenetic reconstruction classified these genes into four evolutionarily conserved subfamilies, namely, the FT-like (two members), TFL1-like (three members), PEBP (three members) and MFT-like (two members) subfamilies (Figure 1), mirroring the subfamily architecture in Arabidopsis, rice, and grapevine [24]. Notably, the expanded TFL1-like subfamily in *B. semperflorens* ‘Super Olympia’ likely originated from lineage-specific whole-genome duplication (WGD) events, a recurrent phenomenon in angiosperms such as *Brassica* and *Malus*, where duplicated paralogs undergo neofunctionalization to adapt to ecological constraints such as shade tolerance [27]. Structural analyses revealed high conservation of motif composition and tertiary structures dominated by random coils, which is consistent with the functional stability of PEBPs across plant lineages. The cis-regulatory elements identified in *BsPEBP* promoters provide molecular insights into *B. semperflorens*’ ‘Super Olympia’ niche-specific adaptations. Light-responsive elements (AE-box, G-box) are enriched in *FT/TFL1-like* genes (*BsPEBP5* and *BsPEBP6*), directly explaining their photoperiod sensitivity in shaded habitats—a key trait enabling continuous flowering under low light. This aligns with *BsPEBP6*’s floral-specific expression (Figure 6), which sustains reproductive development despite canopy cover. Hormone-responsive elements further fine-tune ecological trade-offs: ABRE and GARE motifs in root-specific *BsPEBP3* correlate with its role in ABA/GA-mediated drought response, critical for high-humidity understories, were superficial roots face rapid dehydration. Concurrently, jasmonate (CGTCA-motif) and auxin elements in vegetative *TFL1-like* genes (e.g., *BsPEBP1*) may delay flowering via JA signaling, prioritizing root-shoot biomass allocation in resource-limited niches. Most notably, stress-responsive elements (MYB for drought, LTR for cold) in *BsPEBP3*’s promoter mirror begonia’s native wetland ecology—these motifs likely activate localized root protection against soil waterlogging and chilling, phenomena exacerbated by shaded microclimates. The enrichment of ABRE and GARE motifs in MFT-like promoters aligns with their conserved role in ABA/GA-mediated seed germination [28]. Thus, cis-element diversification not only regulates expression divergence but also embodies adaptive innovations that underpin *B. semperflorens*’ ‘Super Olympia’ resilience in its ecological niche. (Figure 4). These modules suggest that *BsPEBP* genes may act as integrators of environmental and developmental signals. Whereas tissue-specific expression patterns revealed functional divergence: *FT-like BsPEBP6* presented floral organ-enriched expression, whereas TFL1-like members were expressed predominantly in vegetative tissues (Figure 6). This antagonism, with FT-like activators promoting floral induction and TFL1-like repressors maintaining vegetative growth, underlies the continuous-flowering trait of *B. semperflorens* ‘Super Olympia’ (Figure 7). *BsPEBP6* is highly expressed specifically in flower organs (Figure 6), indicating that it is directly involved in the later stage of flower development. As an *FT* homologous gene, it encodes “florigen”, which is a key positive regulatory factor promoting flowering. Similar to *Arabidopsis thaliana FT*, *BsPEBP6* may activate flowering meristematic genes (such as *AP1* and *LFY*) by forming complexes with *FD* (transcription factors), drive the transformation of vegetative meristematic tissues into flowering meristematic tissues, and integrate environmental signals to initiate flowering. *BsPEBP7* is specifically enriched in buds (Figure 6) and may regulate the hormone response elements (such as ABA, JA) in the combination promoter of bud dormancy and activation (Figure 4). It is speculated that by integrating hormone signals (such as ABA inhibiting dormancy), it promotes the rapid transformation of buds into flower buds, shortens the flowering interval, and jointly promotes the continuous flowering of *B. semperflorens* ‘Super Olympia ‘throughout the four seasons.

The retention of duplicated *TFL1-like* genes post-WGD likely enhances regulatory plasticity, enabling fine-tuning of flowering timing in fluctuating environments, as observed in Malus domestica, where duplicated *TFL1* paralogs modulate climate adaptability [25]. Comparative functional analysis highlighted both conservation and innovation. While Arabidopsis *FT* is leaf-localized under long-day conditions, *BsPEBP6* (*FT-like*) shows flower-specific overexpression, suggesting evolutionary rewiring to direct florigen signals directly to floral meristems—a potential adaptation mechanism for perpetual flowering. Silencing *BsTFL1* homologs via CRISPR-Cas9 could increase flowering density [29], as demonstrated in tomato, whereas the overexpression of *BsFT-like* genes might accelerate floral induction in slow-flowering hybrids. While our bioinformatic and expression analyses provide compelling evidence for the roles of *BsPEBP* genes in regulating the continuous-flowering trait of *B. semperflorens* ‘Super Olympia’, it is important to acknowledge that these predictions require functional validation. The absence of transgenic assays (overexpression or silencing of candidate *BsPEBP* genes) represents a limitation of this study. Future work should prioritize functional characterization to confirm the antagonistic roles of FT-like and TFL1-like subfamilies in flowering regulation. Construct overexpression for key candidates. Transform these into *B. semperflorens* ‘Super Olympia’ or model plants to observe phenotypic changes in flowering time and meristem development. Ultimately, this study establishes an evolutionary-functional framework for manipulating flowering time in *Begonia*, with dual benefits for ornamental value enhancement and stress resilience engineering.

## 4. Materials and Methods

### 4.1. Plant Materials

*B. semperforens* ‘Super Olympia’ was planted in the tissue culture room of Henan Agricultural University. The tissue-cultured seedlings were maintained at 23 °C with 14 h of light every day, and the humidity was maintained at approximately 50%.

### 4.2. Identification of PEBP Proteins in B. semperflorens ‘Super Olympia’

To identify *PEBP* candidate genes in *B. semperflorens* ‘Super Olympia’, this study employed two approaches. First, the hidden Markov model (HMM) of the conserved PEBP family domain (Pfam:PF01161) was retrieved from the Pfam database (http://pfam.xfam.org, accessed on 27 March 2025) [30]. We scanned the *B. semperflorens* ‘Super Olympia’ proteome using TBtools to identify candidate genes harboring this domain. Second, *Arabidopsis PEBP* gene data were obtained from the TAIR database (https://www.arabidopsis.org/, accessed on 14 March 2025). Local BLASTP alignment (E-value ≤ 1×10^−5^) was performed via TBtools v2.210 [31] (https://github.com/CJ-Chen/TBtools, accessed on 6 April 2025) to screen homologous candidates. For validation, the candidate genes were further submitted to the NCBI Conserved Domain Database (https://www.ncbi.nlm.nih.gov/Structure/cdd/cdd.shtml, accessed on 22 April 2025) [32] and S-MART (http://smart.embl-heidelberg.de, accessed on 16 April 2025) [33] to confirm the PEBP domain.

### 4.3. Analysis of the Physicochemical Properties of PEBP Family Members in B. semperflorens ‘Super Olympia’

The physicochemical properties of the PEBP proteins, including theoretical pI, molecular weight, and number of amino acids, were predicted using the ExPASy ProtParam tool (http://web.ExPASy.org/protparam, accessed on 11 April 2025) [34]. Subcellular localization was predicted with Cell-PLoc 2.0 online software (http://www.csbio.sjtu.edu.cn/bioinf/Cell-PLoc-2, accessed on 9 March 2025).

### 4.4. Sequence Alignment and Phylogenetic Analysis

Protein amino acid sequences were obtained from Ensembl Plants (https://plants.ensembl.org/index.html, accessed on 19 February 2025). Multiple sequence alignment of *Arabidopsis*, maize, and *Solanum lycopersicum* sequences was performed using ClustalX 11.0 [35]. A phylogenetic tree was constructed with the neighbor-joining (NJ) algorithm in MEGA11.0 [36,37], supported by 1000 bootstrap replicates. The tree was visualized and annotated using the iTOL website (https://itol.embl.de/shared_projects.cgi, accessed on 5 February 2025).

### 4.5. Analysis of Promoter Cis-Acting Elements

The 2000-bp promoter region upstream of the transcription start site of the *BsPEBP* gene was analyzed using the PlantCARE database (http://bioinformatics.psb.ugent.be/webtools/plantcare/html, accessed on 12 February 2025) [38] to identify cis-regulatory elements.

### 4.6. BsPEBP Protein Interaction Network

The protein-protein interaction (PPI) network of BsPEBP was constructed via the STRING website (https://cn.string-db.org, accessed on 19 April 2025) [39].

### 4.7. Phylogenetic Evolution and Conserved Motif Analysis

The phylogenetic tree was constructed using MEGA 11.0 with the following nine parameters: multiple sequence alignment by the MUSCLE method; tree reconstruction using the neighbor-joining algorithm with 1000 bootstrap replicates. Conserved motif analysis of amino acid sequences was performed via the MEME suite (http://meme-suite.org/meme/tools/meme, accessed on 2 February 2025) [40], with the number of motifs set to 10, and visualization was conducted with TBtools v2.210 software.

### 4.8. Protein Secondary and Tertiary Structure Prediction

The secondary structure of the *BsPEBP* gene was predicted using the online tool SOPMA, and the proportions of alpha helices, beta turns, extended strands, and random coils in the overall structure were analyzed. SWISS-MODEL (https://swissmodel.expasy.org, accessed on 7 March 2025) [41,42,43,44,45] was used to perform homology modeling and predict the tertiary structure of the BsPEBP protein.

### 4.9. RNA Extraction, cDNA Synthesis, and qRT-PCR Experiments

Experimental consumables (centrifuge tubes and pipette tips) were sterilized at high temperature to eliminate RNases. The samples were retrieved from the ultralow temperature freezer and placed in a liquid nitrogen tank. RNA was extracted via a kit (Novizan Biotechnology Co., Nanjing, China) following the manufacturer’s instructions. cDNA was synthesized via a reverse transcription kit (Luohe Biotechnology Co., Hangzhou, China) according to the manufacturer’s protocol and stored at −20 °C. The qRT-PCR system was prepared following the instructions of the TOROBlue qRT PreMix with gDNA Eraser 2.0 kit (Luohe Biotechnology Co., Hangzhou, China), and fluorescence quantification was performed using TOROGreen qPCR Master Mix (Luohe Biotechnology Co., Hangzhou, China). The qRT-PCR experiments were conducted with primers (Table S4). Moreover, Bs18S (GenBank: KJ959633) was used as a reference gene. Each sample contained three individual plant replicates, and the experiment was repeated three times. Three technical replicates of the qRT-PCR experiments were performed. Relative expression data were processed using the 2^−ΔΔCt^ [46] method in Microsoft Excel, visualized through GraphPad Prism 9.5 software, and statistical significance was evaluated by Student’s *t*-test.

## 5. Conclusions

Through genome-wide analysis, we identified ten *PEBP* genes (designated *BsPEBP1*-*BsPEBP10*) in *B. semperflorens* ‘Super Olympia’, which were phylogenetically classified into four distinct subfamilies. We performed comprehensive genomic analyses, including phylogenetic reconstruction, functional classification, gene structure characterization, motif analysis, chromosomal mapping, and identification of genomic loci of the *PEBP* gene family in this species. Our investigation revealed stage-specific expression patterns of *BsPEBPs* during floral development. Functional analysis showed that FT-like subfamily members promote flowering, whereas *TFL1-like* genes repress it. These findings provide significant insights into the molecular mechanisms underlying the regulatory roles of *PEBP* genes in floral development of *B. semperflorens* ‘Super Olympia’.

## Data Availability

This study includes all collected and analyzed data in the main manuscript and supplementary documents. Any additional inquiries may be directed to the corresponding author.

## Supplementary Materials

The following supporting information can be downloaded at: https://www.mdpi.com/article/10.3390/ijms26136291/s1.
