# Supplementary material for: Genome-Wide Identification and Functional Analysis of the PEBP Gene Family in Begonia semperflorens ‘Super Olympia’ Reveal Its Potential Role in Regulating Flowering
Source: Int J Mol Sci. 2025 Jun 29;26(13):6291. doi: 10.3390/ijms26136291 (PMC12249742; doi:10.3390/ijms26136291)
Supplement: Supplementary file 1 [file ijms-26-06291-s001.zip › Figure S1-S3.pdf]

```
BsPEBP1       ....MSRTMEPLIVGRVVGEVVDIFNPTVKMNVIYNSNKQVANGHELMPSAVISKPRVDIGGDDLRSAYTLIMTDPDAPS    76
BsPEBP2       ..............MANASDDFRLVSPAIDHDGRLP...RKYLWEGQGATKDVSPPLEWYNLPEGTKTLALVVQDIDAPD    63
BsPEBP3       MEGSNHQSKKSKSAMANASDDFRLVSPAIDHDGRLP...RKYLWEGQGATKDVSPPLEWYNLPEGTKTLALVVQDIDAPD    77
BsPEBP4       ....MHFWLGPLACGPLLHHGQPSWHPNIAPRGCVFT.TEEVLMEGQGMTKDVSPPLEWYNLPAGTKTLALVVQDIDASD    75
BsPEBP5       ....MERTVEPLLVGRVIGDVLDYFIPSIKLSVTFNS.KKVFNGHEFLPSTVAAKPRVEIQGGDLRSFFTLVMTDPDVPG    75
BsPEBP6       ....MPRDRDPLVVGRVIGDVLDPFIRSISLRVCYTS.KDVNNGCELKPSQVVNQPRVEIGGDDLRTFYTLVMVDPDAPS    75
BsPEBP7       ....MRRDRDPLVVGRVIGDVLDPFIRSISLRVCHNS.KEVNNGCELKPSQVVSQPRVEVGGDDLRTFFTLVMVDPDAPS    75
BsPEBP8       ....MATSVDPLVVGRVIGDVVDMFVPVANLSVYFGA.KHVTNGCDIKPSLAADPPRFIVPG.HVDDLYTLILTDPDAPS    74
BsPEBP9       ....MSASVDPLVVGKVIGEVVDMFIPTGNMSVYFGN.KQVTNGCHIKPSTATDPPRISISG.HSHDLYTLVMTDPDAPS    74
BsPEBP10      ....MANKADPLVVGRVIGDVVDYFTPTVKMTVTYNSSARVYNGQEFLPSPVSIKPKVEVHGGDLRSFFTLVMTDPDVPG    76
Consensus                                                            p              l   d d

BsPEBP1       PSDPCLREHLNWMVTDIPGTTN.ASFGREIISYETPKPMVGIHRYVFVLFKQKGRQ.TVRA..PSSRDHFCTRTFAEANG    152
BsPEBP2       PDGP.IVPWTVWVVVNIPPELKGLPEGFSGKGEEAGGEYRGIQEGNNDMKVPGWRGPKLPS..HGHRFEFKLFALDDELR    140
BsPEBP3       PDGP.IVPWTVWVVVNIPPELKGLPEGFSGKGEEAGGEYRGIQEGNNDMKVPGWRGPKLPS..HGHRFEFKLFALDDELR    154
BsPEBP4       PDGS.IVPWTVWVVVNISPKLKGLPEGFSGKGEEYVER.........................................    112
BsPEBP5       PSDPYLREHLHWIVTDIPGTTD.ATFGREVVSYENPKPNIGIHRFVFVVFKQKSRL.SVNP.RSSSRDYFNTRSFAEEHD    152
BsPEBP6       PSDPNLREYLHWLVTDIPATTG.ASFGQEVVCYESPRPTMGIHRFVFVLFRQLGRQ.TVYA..PGWRQNFNTKDFAELYH    151
BsPEBP7       PSDPNLREYLHWLVTDIPATTG.ATFGQEVVCYESPRPTMGIHRFVFVLFRQLGRQ.TVYA..PGWRQNFSTKDFAELYN    151
BsPEBP8       PSEPHMREWVHWIVVDIPGGAS.PSRGKEILPYIGPRPPIGIHRYILLLFKQKGPL.TIVNQPPPSRANFNTRHFTHHLD    152
BsPEBP9       PSEPTLREWVHWVVVDIPGGTN.PNGGEEIVPYMGPQPPVGIHRYVVVLFKQRSQVGSLVNRLIEGRSYFSTRLFARQLD    153
BsPEBP10      PSDPYLKEHLHWMVTDIPGTTS.ATFGKEIVKYEMPRPIIGIHRFVFILYEQKRRE.TVKPPNLSTRDGFSSRKFAEENN    154
Consensus     p          w v  i         g

BsPEBP1       LSLPVAAVYFNAQRETAA..RRR....                                                        173
BsPEBP2       LGNKATKEKLYDAIEGHVLGEATLMAV                                                        167
BsPEBP3       LGNKATKEKLYDAIEGHVLGEATLMAV                                                        181
BsPEBP4       ...........................                                                        112
BsPEBP5       LGLPVAAVYFNAQRETAA..RRR....                                                        173
BsPEBP6       LGLPVAALYYNCQRESGSGGRRR....                                                        174
BsPEBP7       LGLPVAAVYYNCQRESGSGRRRR....                                                        174
BsPEBP8       LGLPVAATYFNSQKEPAA..KKH....                                                        173
BsPEBP9       LGLPVAATYFHSQKEPAA..KRR....                                                        174
BsPEBP10      LGLPVAAVFFNAQRETAS..RKR....                                                        175
Consensus
```

Figure S1. Sequence comparison of PEBP family proteins.

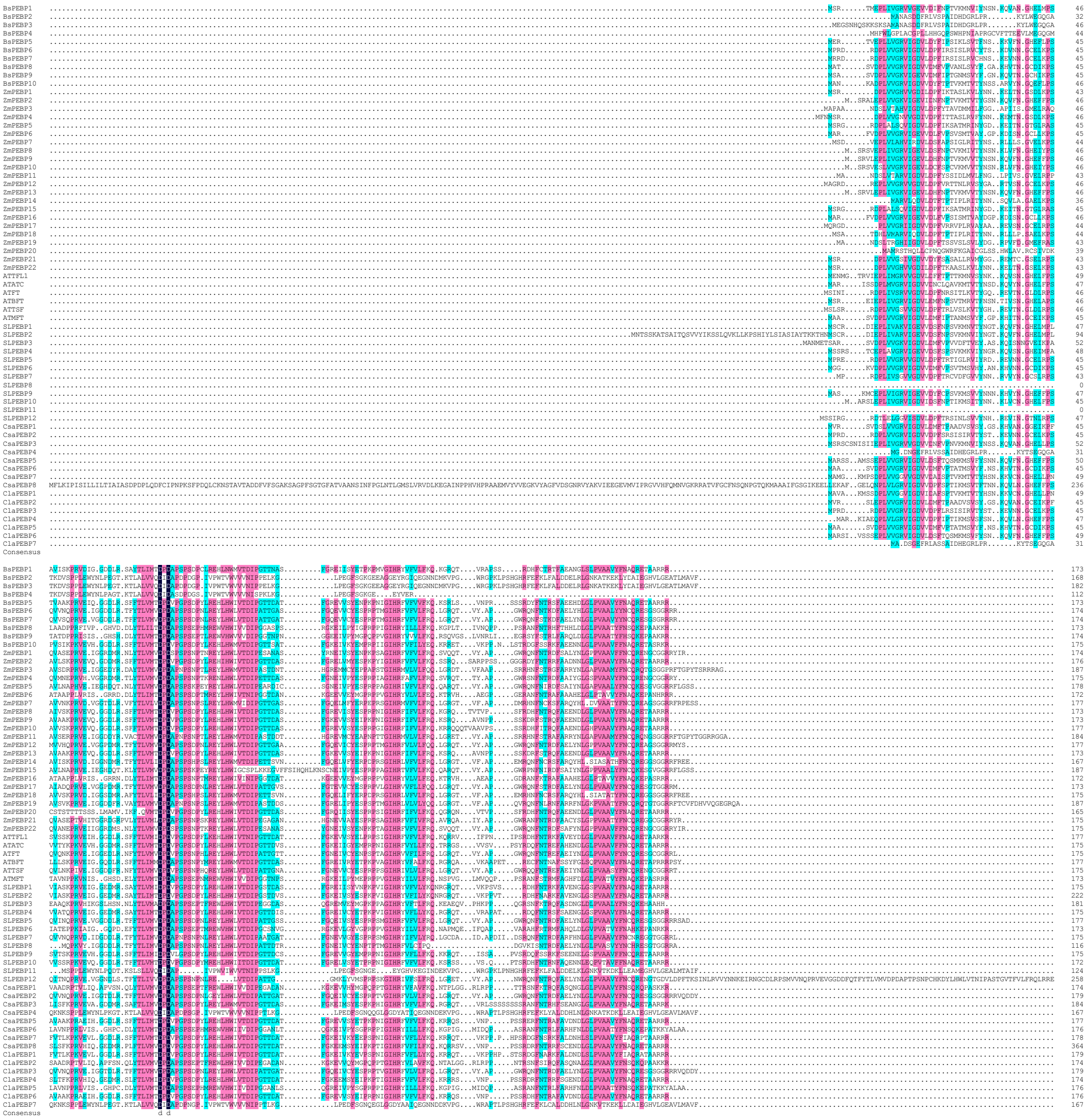

Figure S2. Multiple sequence alignment of *Arabidopsis thaliana*, *Solanum lycopersicum*, *Zea mays*, *B. semperflorens* ‘Super Olympia’, Citrullus *lanatus*, and *Cucumis sativus*.

```
BsPEBP1    MSR....TMEPLIVGRVVGEVVDIFNPTVKMNVIYNSNKQVANGHELMPSAVISKPRVDIGGDDLRSAYTLIMTDPDAPS    76
BsPEBP5    ME....RTVEPLLVGRVIGDVLDYFIPSIKLSVTFNS.KKVFNGHEFLPSTVAAKPRVEIQGGDLRSFFTLVMTDPDVPG    75
BsPEBP10   MANK....ADPLVVGRVIGDVVDYFTPTVKMTVTYNSSARVYNGQEFLPSPVSIKPKVEVHGGDLRSFFTLVMTDPDVPG    76
ATTFL1     MENMGTRVIEPLIMGRVVGDVLDFFTPTTKMNVSYNK.KQVSNGHELFPSSVSSKPRVEIHGGDLRSFFTLVMIDPDVPG    79
ATBFT      MSR....EIEPLIVGRVIGDVLEMFNPSVTMRVTFNSNTIVSNGHELAPSLLLSKPRVEIGGQDLRSFFTLIMMDPDAPS    76
ATATC      MARIS...SDPLMVGRVIGDVVDNCLQAVKMTVTYNSDKQVYNGHELFPSVVTYKPKVEVHGGDMRSFFTLVMTDPDVPG    77
Consensus  m         pl  grv g v           v  n    v ng e  ps    kp v   g d rs  tl m dpd p

BsPEBP1    PSDPCLREHLNWMVTDIPGTTNASFGREIISYETPKPMVGIHRYVFVLFKQKGRQTVR..APSSRDHFCTRTFAEANGLS    154
BsPEBP5    PSDPYLREHLHWIVTDIPGTTDATFGREVVSYENPKPNIGIHRFVFVVFKQKSRLSVNP.RSSSRDYFNTRSFAEEHDLG    154
BsPEBP10   PSDPYLKEHLHWMVTDIPGTTSATFGKEIVKYEMPRPIIGIHRFVFILYEQKRRETVKPPNLSTRDGFSSRKFAEENNLG    156
ATTFL1     PSDPFLKEHLHWIVTNIPGTTDATFGKEVVSYELPRPSIGIHRFVFVLFRQKQRRVIFP.NIPSRDHFNTRKFAVEYDLG    158
ATBFT      PSNPYMREYLHWMVTDIPGTTDASFGREIVRYETPKPVAGIHRYVFALFKQRGRQAVKA.APETRECFNTNAFSSYFGLS    155
ATATC      PSDPYLREHLHWIVTDIPGTTDVSFGKEIIGYEMPRPNIGIHRFVYLLFKQTRRGSVVS.VPSYRDQFNTREFAHENDLG    156
Consensus  ps p   e l w vt ipgtt   fg e   ye p p  gihr v     q  r          r  f    f     l

BsPEBP1    LPVAAVYFNAQRETAARRR..                                                                173
BsPEBP5    LPVAAVYFNAQRETAARRR..                                                                173
BsPEBP10   LPVAAVFFNAQRETASRKR..                                                                175
ATTFL1     LPVAAVFFNAQRETAARKR..                                                                177
ATBFT      QPVAAVYFNAQRETAPRRRPS                                                                176
ATATC      LPVAAVFFNCQRETAARRR..                                                                175
Consensus   pvaav fn qreta r r
```

(A). Multiple sequence alignment of *Arabidopsis thaliana TFL1-like, and B. semperflorens* 'Super Olympia' *TFL1-like*.

```
BsPEBP8    MATSVDPLVVGRVIGDVVDMFVPVANLSVYFGAKHVTNGCDIKPSLAADPPRFIVPGHVDDLYTLIITDPDAPSPSEPHM    80
BsPEBP9    MSASVDPLVVGKVIGEVVDMFIPTGNMSVYFGNKQVTNGCHIKPSTATDPPRISISGHSHDLYTLVMTDPDAPSPSEPTL    80
ATMFT      MAASVDPLVVGRVIGDVLDMFIPTANMSVYFGPKHITNGCEIKPSTAVNPPKVNISGHSDELYTLVMTDPDAPSPSEPNM    80
Consensus  m  svdplvvg vig v dmf p  n svyfg k  tngc ikps a  pp     gh   lytl  tdpdapspsep

BsPEBP8    REWVHWIVVDIPGGASPSRGKEILPYIGPRPPIGIHRYILLLFKQKGPL.TIVNQPPPSRANFNTRHFTHHLDLGLPVAA    159
BsPEBP9    REWVHWVVVDIPGGTNPNGGEEIVPYMGPQPPVGIHRYVVVLFKQRSQVGSLVNRLIEGRSYFSTRLFARQLDLGLPVAA    160
ATMFT      REWVHWIVVDIPGGTNPSRGKEILPYMEPRPPVGIHRYILVLFRQNSPVGLMVQQP.PSRANFSTRMFAGHFDLGLPVAT    159
Consensus  rewvhw vvdipgg  p  g ei py  p pp gihry   lf q       v      r  f tr f    dlglpva

BsPEBP8    TYFNSQKEPAAKK                                                                        172
BsPEBP9    TYFHSQKEPAAKR                                                                        173
ATMFT      VYFNAQKEPASRR                                                                        172
Consensus   yf  qkepa
```

(B). Multiple sequence alignment of *Arabidopsis thaliana* MFT-like, *and B. semperflorens* 'Super Olympia' MFT-like.

```
BsPEBP6    MPRD.RDPLVVGRVIGDVLDPFIRSISLRVCYTSKDVNNGCELKPSQVVNQPRVEIGGDDLRTFYTLVMVDPDAPSPSDP    79
BsPEBP7    MRRD.RDPLVVGRVIGDVLDPFIRSISLRVCHNSKEVNNGCELKPSQVVSQPRVEVGGDDLRTFFTLVMVDPDAPSPSDP    79
ATFT       MSINIRDPLIVSRVVGDVLDPFNRSITLKVTYGQREVTNGLDLRPSQVQNKPRVEIGGEDLRNFYTLVMVDPDVPSPSNP    80
ATTSF      MSLSRRDPLVVGSVVGDVLDPFTRLVSLKVTYGHREVTNGLDLRPSQVLNKPIVEIGGDDFRNFYTLVMVDPDVPSPSNP    80
Consensus  m    rdpl v  v gdvldpf r   l v      v ng  l psqv   p ve gg d r f tlvmvdpd psps p

BsPEBP6    NLREYLHWLVTDIPATTGASFGQEVVCYESPRPTMGIHRFVFVLFRQLGRQTVYAPGWRQNFNTKDFAELYHLGLPVAAL    159
BsPEBP7    NLREYLHWLVTDIPATTGATFGQEVVCYESPRPTMGIHRFVFVLFRQLGRQTVYAPGWRQNFSTKDFAELYNLGLPVAAV    159
ATFT       HLREYLHWLVTDIPATTGTTFGNEIVCYENPSPTAGIHRVVFILFRQLGRQTVYAPGWRQNFNTREFAEIYNLGLPVAAV    160
ATTSF      HQREYLHWLVTDIPATTGNAFGNEVVCYESPRPPSGIHRIVLVLFRQLGRQTVYAPGWRQQFNTREFAEIYNLGLPVAAS    160
Consensus    reylhwlvtdipattg  fg e vcye p p  gihr v  lfrqlgrqtvyapgwrq f t  fae y lglpvaa

BsPEBP6    YYNCQRESGSGGRR                                                                       173
BsPEBP7    YYNCQRESGSGRRR                                                                       173
ATFT       FYNCQRESGCGGRR                                                                       174
ATTSF      YFNCQRENGCGGRR                                                                       174
Consensus    ncqre g g rr
```

(C). Multiple sequence alignment of *Arabidopsis thaliana* FT-like, *and B. semperflorens* 'Super Olympia' FT-like.

Figure S3. Multiple sequence alignment for PPI network prediction.
