# Supplementary material for: Genome-Wide Identification and Functional Analysis of the PEBP Gene Family in Begonia semperflorens ‘Super Olympia’ Reveal Its Potential Role in Regulating Flowering
Source: Int J Mol Sci. 2025 Jun 29;26(13):6291. doi: 10.3390/ijms26136291 (PMC12249742; doi:10.3390/ijms26136291)
Supplement: Supplementary file 1 [file ijms-26-06291-s001.zip › Table S1.pdf]

Table S1：Gene information and physicochemical properties of *PEBP* genes in *B. semperflorens* 'Super Olympia'

Gene name	Number of aminoacid（aa）	Molecular weight (Da)	Theoretical PI	Instability index	Lipid Soluble index	Grand average of hydropathicity	Subcellular localization
*BsPEBP*1	173	19294.11	9.26	40.31	78.84	-0.272	Cytoplasm
*BsPEBP*2	168	18530.96	4.9	32.28	81.25	-0.456	Nucleus
*BsPEBP*3	182	20031.61	5.26	32.98	75.55	-0.56	Nucleus
*BsPEBP*4	112	12276.07	4.98	47.6	85.18	-0.123	Nucleus
*BsPEBP*5	173	19610.31	8.96	37.96	82.14	-0.279	Cytoplasm
*BsPEBP*6	174	19708.42	7.68	47.21	81.09	-0.33	Nucleus
*BsPEBP*7	174	19760.5	8.78	52.89	79.94	-0.344	Nucleus
*BsPEBP*8	173	18987.97	7.95	32.95	94.05	-0.076	Cytoplasm、Nucleus.
*BsPEBP*9	174	19093.86	7.92	48.2	84.43	-0.169	Cytoplasm
*BsPEBP*10	175	19717.64	9.33	41.29	76.74	-0.319	Cytoplasm、Nucleus.
