# Supplementary material for: Genome-Wide Identification and Functional Analysis of the PEBP Gene Family in Begonia semperflorens ‘Super Olympia’ Reveal Its Potential Role in Regulating Flowering
Source: Int J Mol Sci. 2025 Jun 29;26(13):6291. doi: 10.3390/ijms26136291 (PMC12249742; doi:10.3390/ijms26136291)
Supplement: Supplementary file 1 [file ijms-26-06291-s001.zip › Table S3.pdf]

Table S3：Predicted secondary structure of BsPEBP protein

Gene name	Alpha helix	Beta turn	Extended strand	Random coil
*BsPEBP*1	15.03	0	26.01	58.96
*BsPEBP*2	14.88	0	17.86	67.26
*BsPEBP*3	13.19	0	16.48	70.33
*BsPEBP*4	0	0	20.54	79.46
*BsPEBP*5	15.03	0	25.43	59.54
*BsPEBP*6	13.22	0	26.44	60.34
*BsPEBP*7	14.37	0	25.29	60.34
*BsPEBP*8	14.45	0	25.43	60.12
*BsPEBP*9	15.52	0	24.71	59.77
*BsPEBP*10	14.29	0	24.57	61.14
