# Supplementary material for: Genome-Wide Identification and Functional Analysis of the PEBP Gene Family in Begonia semperflorens ‘Super Olympia’ Reveal Its Potential Role in Regulating Flowering
Source: Int J Mol Sci. 2025 Jun 29;26(13):6291. doi: 10.3390/ijms26136291 (PMC12249742; doi:10.3390/ijms26136291)
Supplement: Supplementary file 1 [file ijms-26-06291-s001.zip › Table S4.pdf]

Table S4: Primers used in this study

Gene name	Forward primer (5'-3')	Reverse primer (5'-3')
*BsPEBP*1	CCCGAGCGATCCTTGCTT	TTGGCTTTGGCGTCTCGT
*BsPEBP*2	AAAGGGGAAGAAGCCGGC	CCCAGGCGCAACTCATCA
*BsPEBP*3	TTGTGGGAAGGTCAAGGCGC	TCATCCAAAGCAAAGAGCTT
*BsPEBP*4	AATCTACCGGCGGGGACT	AAATCCCTCCGGCAAGCC
*BsPEBP*5	GCCATCAACTGTTGCCGC	AGGATCACTCGGACCAGGA
*BsPEBP*6	TTTCGGCAGCTCGGAAGG	CTGACCCGCTCTCCCTCT
*BsPEBP*7	CCTTCGGGCAAGAGGTGG	GGGCGTATACCGTCTGCC
*BsPEBP*8	CATAGGGCCACGACCACC	GGTGAAGTGGCGGGTGTT
*BsPEBP*9	CCGGCTAATCGAGGGTCG	TGGAAGTAAGTGGCGGCG
*BsPEBP*10	AGCGGATCCTCTGGTCGT	GGAAGAAACTCCTGCCCGT
Bs18S	GCTACCACATCCAAGGAAGG	CAATGGATCCTCGTTAAGGG
